# Expression of long non‐coding RNA LUCAT1 in patients with chronic obstructive pulmonary disease and its potential functions in regulating cigarette smoke extract‐induced 16HBE cell proliferation and apoptosis

**DOI:** 10.1002/jcla.23823

**Published:** 2021-06-14

**Authors:** Shan Zhao, Chunyan Lin, Tao Yang, Xiaoyu Qian, Junjie Lu, Jing Cheng

**Affiliations:** ^1^ Department of Clinical Laboratory Affiliated Yixing People’s Hospital Jiangsu University Wuxi China; ^2^ Department of Blood Transfusion The First Affiliated Hospital of Soochow University Suzhou China; ^3^ Department of Critical Care Medicine Affiliated Yixing People’s Hospital Jiangsu University Wuxi China

**Keywords:** 16HBE, chronic obstructive pulmonary disease, cigarette smoke extract, lncRNA LUCAT1, miR‐181a‐5p

## Abstract

**Background:**

Chronic obstructive pulmonary disease (COPD), characterized by persistent airflow limitation, was a disease mediated by a combination of inflammatory factors, immune cells, and immune mediators. COPD was an inflammatory and autoimmune disease involving T‐lymphocytes triggered by cigarette smoke and other factors that progressively affected the bronchi, lung parenchyma, and pulmonary blood vessels. LncRNAs were reported to be implicated in COPD pathogenesis and development.

**Methods:**

Non‐smokers, smokers (non‐COPD), and COPD patients were randomly selected in an established COPD surveillance cohort. Demographic and clinical information of all subjects were collected. Pulmonary function was measured by post‐bronchodilator testing. qRT‐PCR and ELISA assays were performed to detect the expression levels of lncRNA LUCAT1, miR‐181a‐5p, and inflammatory cytokines. An in vitro exposure model was constructed using cigarette smoke extract (CSE)‐induced human bronchial epithelial (16HBE) cells. The dual‐luciferase reporter and RNA pull‐down assays were used to detect the binding relationship between lncRNA LUCAT1 and miR‐181a‐5p; meanwhile, Spearman's correlation assay was used to verify the correlation between lncRNA LUCAT1 and miR‐181a‐5p. Afterward, the lncRNA LUCAT1 silencing plasmid was constructed and co‐transfected with a miR‐181a‐5p inhibitor to evaluate the effects on CSE‐induced 16HBE cell proliferation and apoptosis. Finally, a Western blot assay was utilized to determine the mechanism of lncRNA LUCAT1/miR‐181a‐5p/Wnt/β‐catenin axis in COPD.

**Results:**

LncRNA LUCAT1 was upregulated in the serums of COPD patients. Correlation analysis further confirmed the strong correlation between LUCAT1 expression and inflammatory cytokines IL‐1β, IL‐6, and TNF‐α. Receiver operating characteristic (ROC) analysis verified the potential of LUCAT1 in COPD diagnosis. After treatment with CSE, LUCAT1 was significantly increased while its target miR‐181a‐5p was decreased in 16HBE cells. Cell proliferation and apoptosis assays showed that LUCAT1 silencing alleviated CSE’s effects on 16HBE cell proliferation and apoptosis. Mechanically, rescue assays demonstrated that miR‐181a‐5p inhibition could partially counteract the impact of LUCAT1 on COPD progression through the Wnt/β‐catenin pathway.

**Conclusions:**

LncRNA LUCAT1 may be a valuable indicator for differentiating COPD. Moreover, LncRNA LUCAT1/miR‐181‐5p/Wnt/β‐catenin axis behaved as a critical role in COPD development, shedding new sights for clinical treatment.

## Graphical abstract

Chronic obstructive pulmonary disease (COPD) was a chronic, non‐homogeneous lung disease characterized by persistent excessive inflammation that led to tissue remodeling, alveolar damage, airflow limitation, and accelerated lung function decline. The clinical manifestations of COPD include chronic cough, coughing sputum, dyspnea, and acute exacerbation in the presence of infections and other triggering factors. Pulmonary function tests were the primary means of confirming the diagnosis of COPD (Respiratory Care, 57, 2012 and 165). Appropriate drug therapy can effectively relieve symptoms, reduce the number and frequency of acute exacerbations, improve exercise tolerance, and improve quality of life. Cigarette smoking was a significant risk factor for COPD. Inhaled cigarette smoke first encountered respiratory epithelial cells, causing the release of pro‐inflammatory mediators such as interleukin (IL)‐1, IL‐6, and tumor necrosis factor (TNF)‐α. Although inflammation‐based airway injury was recognized as a core feature of COPD, the underlying mechanism was unclear. From the results, we found that lncRNA LUCAT1 was upregulated in the serums of COPD patients. Correlation analysis further confirmed the strong correlation between LUCAT1 expression and inflammatory cytokines IL‐1β, IL‐6, and TNF‐α. Receiver operating characteristic (ROC) analysis verified the potential of LUCAT1 in COPD diagnosis. After treatment with CSE, LUCAT1 was significantly increased, while its target miR‐181a‐5p was decreased in 16HBE cells. Cell proliferation and apoptosis assays showed that LUCAT1 silencing alleviated CSE’s effects on 16HBE cell proliferation and apoptosis. Mechanically, rescue assays demonstrated that miR‐181a‐5p inhibition could partially counteract the impact of LUCAT1 on COPD progression through the Wnt/β‐catenin pathway. In sum, lncRNA LUCAT1 may be a valuable indicator for differentiating COPD. Moreover, lncRNA LUCAT1/miR‐181‐5p/Wnt/β‐catenin axis behaved as a critical role in COPD development, shedding new sights for clinical treatment.

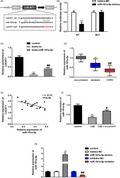

## 1 INTRODUCTION

Chronic obstructive pulmonary disease (COPD) was a chronic, non‐homogeneous lung disease characterized by persistent excessive inflammation that led to tissue remodeling, alveolar damage, airflow limitation, and accelerated lung function decline.^1^ The clinical manifestations of COPD include chronic cough, coughing sputum, dyspnea, and acute exacerbation in the presence of infections and other triggering factors.^2^ Pulmonary function tests were the primary means of confirming the diagnosis of COPD.^3^ Appropriate drug therapy can effectively relieve symptoms, reduce the number and frequency of acute exacerbations, improve exercise tolerance, and improve quality of life. Cigarette smoking was a significant risk factor for COPD.^4^ Inhaled cigarette smoke first encountered respiratory epithelial cells, causing the release of pro‐inflammatory mediators such as interleukin (IL)‐1, IL‐6, and tumor necrosis factor (TNF)‐α.^5^, ^6^, ^7^ Although inflammation‐based airway injury was recognized as a core feature of COPD, the underlying mechanism was unclear.

LncRNAs are a class of non‐coding RNAs (ncRNAs), which have been found to play regulatory roles in cell normal physiological homeostasis and lung disease development.^8^, ^9^, ^10^ Simultaneously, its importance in pulmonary pathology contributed to the development of lncRNA‐based therapeutic strategies and biomarker tools.^11^ Although several studies have shown that smoking affected lncRNA expressions and played a vital role in the development and progression of COPD,^12^, ^13^, ^14^ its exact function remained to be further investigated. Therefore, in‐depth studies on the roles of lncRNAs in smoking‐induced COPD pathogenesis and development were of great theoretical significance and practical value in elucidating the molecular mechanisms of smoking‐induced COPD and contributing to therapeutic targets.

## 2 MATERIALS AND METHODS

### 2.1 Subjects recruitment and sample collection

Patients diagnosed with COPD, those who do not have COPD but are current smokers (smokers), and those who do not have COPD and have never smoked (non‐smokers) were recruited from the First Affiliated Hospital of Soochow University between April 2017 and December 2019. All survey respondents gave informed consent. Ethics Committee of the First Affiliated Hospital of Soochow University approved this study. Demographic information and clinical information were presented in Table 1.

**TABLE 1:** The clinical features of all subjects

Features	Non‐smokers (*N* = 72)	Smokers (*N* = 78)	COPD (*N* = 70)
Gender			
Male	45	56	51
Female	27	22	19
Age (years)	67.5 ± 6.5	65.9 ± 7.2	66.9 ± 6.8
Hypertension (%)	29.2	39.7	40.0
Diabetes mellitus (%)	16.7	21.8	21.4
Smoking duration (years)	0	28.5 ± 5.6	31.2 ± 4.2
COPD history			
Yes	/	/	28
No	/	/	42
BMI (kg/m^2^)	25.1 ± 1.0	24.8 ± 2.2	25.6 ± 1.8
FEV1 (L)	3.3 ± 0.5	2.8 ± 0.2	2.1 ± 0.3
FEV1%	94.1 ± 3.8	72.8 ± 6.8	43.5 ± 5.2
FEV1/FVC ratio	82.7 ± 5.2	76.9 ± 4.5	58.2 ± 6.3
IL‐1β	3.2 ± 1.1	6.3 ± 2.2	12.6 ± 3.5
IL‐6	9.8 ± 3.4	15.7 ± 5.0	22.9 ± 6.8
TNF‐α	12.5 ± 5.7	13.2 ± 4.9	18.1 ± 7.3

Chronic obstructive pulmonary disease diagnostic criteria: the ratio of first, second exertional expiratory volume (FEV) to forced lung capacity (FVC) after bronchodilation testing was less than 70%, following the Global Initiative for Chronic Obstructive Pulmonary Disease. Exclusion criteria: patients with comorbidities, including asthma, interstitial lung disease, heart failure, or neuromuscular disease; any respiratory infection or glucocorticoid or antibiotic administration in the month before the study.

Five millilitres of venous blood was collected from all subjects. All samples were rested for 30 min at room temperature, centrifuged at 1000 *g* at 4°C, and stored at −80°C until use. A trained physician performed pulmonary function measurements. All subjects inhaled 400 µg of salbutamol aerosol, rested for 15 min, and then inhaled deeply through the mouthpiece until complete, with a sudden exhalation. Subjects should continue to exhale actively and for as long as possible.

### 2.2 ELISA assay

Dilute the standards according to the ELISA kit instructions. 100 µl of biotinylated antibody was added to each well and incubated at 37°C for 60 min. After washing for three times, 100 µl of enzyme binding solution was added to each well and incubated at 37°C for 30 min after lamination. Then, 50 µl of termination solution was added to each well, and the OD value of each well was immediately measured by an enzyme meter.

### 2.3 Preparation of cigarette smoke extracts (CSE)

Refer to a previous study,^15^ a standard cigarette was connected to an extraction device and lit. The cigarette smoke was extracted by the extraction device at a constant speed and dissolved in 37°C pre‐warmed serum‐free MEM medium. For quality control, the absorbance values at 320 nm (A320) and 540 nm (A540) were measured using a spectrophotometer, and CSE solutions with △OD (A320‐A540) in the range of 0.9 and 1.2 were considered acceptable. The final CSE solution was used as a 1 mg/ml CSE master mix, dispensed, and stored in a −80°C refrigerator, protected from light.

### 2.4 Cell culture and CSE treatment

The 16HBE cell line and H293K cell line used in this experiment were purchased from the Institute of Cell Biology, Shanghai Institute of Life Sciences, Chinese Academy of Sciences. After the cells were slowly rinsed with preheated PBS at 37°C, the cells were digested with trypsin digestive medium and centrifuged. Then, cells were incubated in a 37°C incubator with 5% CO_2_. The next day, 16HBE cells were treated with fresh culture medium containing 20 µg/ml CSE, and total RNA or protein was collected for subsequent experiments.

### 2.5 qRT‐PCR analysis

Total RNA was extracted from serums and cells using TRIzol reagent (Invitrogen) under the instructions. Reverse transcription was performed with a PrimeScript RT reagent kit (Takara). Then, PCR analysis was conducted on an ABI7500 PCR machine (Thermo Fisher Scientific, Inc.) using SYBR Green RealTime PCR Kit (Takara) following the manufacturer's protocol. The thermal cycle conditions were as follows: 95°C for 5 min, 40 cycles of 95°C for 1 min, and 64°C for 30 s. Finally, equation 2^−△△CT^ method was utilized for quantifying relative expressions of LUCAT1 and miR‐181a‐5p. U6 functioned as an endogenous control. The primers sequences were as follows: LUCAT1 forward, 5′‐GCTCGGATTGCCTTAGACAG‐3′, and reverse, 5′‐GGGTGAGCTTCTTGTGAGGA‐3′; miR‐181a‐5p forward, 5′‐ACACTCCAGCTGGGAACATTCAACGCTGTCGG‐3′, and revers, 5′‐TGGTGTCGTGGAGTCGA‐3′; U6 forward, 5′‐CTCGCTTGGGCAGCACA‐3′, and reverse, 5′‐AACGCTTCACGAATTTGCGT‐3′.

### 2.6 Dual‐luciferase reporter assay

H293K cells were seeded in 24‐well plates with a density of 2 × 10^5^ cells/well and incubated for 24 h at room temperature in a humidified incubator with 5% CO_2_. Then, wild type (WT) and mutant type (MUT) of LUCAT1 were inserted into the pmirGLO vector to construct pmirGLO‐LUCAT1‐WT and pmirGLO‐LUCAT1‐MUT plasmids. Following the instructions on the detection kit, H293 cells were transfected with pmirGLO‐LUCAT1‐WT or pmirGLO‐LUCAT1‐MUT and mimics‐NC or miR‐181a‐5p mimics. After 24 h of incubation, luciferase activity was evaluated by SuperLight™ Luciferase Reporter Gene Assay Kit (BioAssay Systems) and normalized to the corresponding Renilla luciferase activity.

### 2.7 RNA pull‐down assay

After treatment with CSE, cell lysates were incubated with a miR‐181a‐5p probe or Oligo probe following the instructions. Then, cells were incubated with streptavidin‐coated magnetic beads as described before. Finally, qRT‐PCR analysis was performed to evaluate the mRNA enrichment.

### 2.8 Plasmid construction and cell transfection

Invitrogen Co., synthesized LUCAT1 small interfering (si‐LUCAT1) and negative control (si‐NC). miR‐181a‐5p mimics, miR‐181a‐5p inhibitor, mimics‐NC, and inhibitor‐NC were designed by GenePharma. Then, 16HBE cells were transfected with 10 mM miR‐181a‐5p mimics, miR‐181a‐5p inhibitor, mimics‐NC, and inhibitor‐NC or 40 mM si‐LUCAT1 and si‐NC according to the instructions. Transfection was conducted at 48 h incubation.

### 2.9 Cell viability

Cell viability was evaluated by cell counting kit‐8 (CCK‐8; Dojindo) kit according to the instructions. In brief, treated and transfected cells were seeded in a 96‐well plate with a density of 5 × 10^4^ cells/well. At 12, 24, and 48 h, 10 μl of CCK‐8 solution was added to each well and incubated for 2 h before absorbance detection. Each group's absorbance was measured on a microplate reader (BioTech) at 450 nm.

### 2.10 Cell apoptosis

At 48 h post‐transfection, cells were collected and stained with Annexin V‐FITC/PI detection kit (BD Bioscience) following the manufacturer's protocol. After 20 min of incubation at room temperature under 5% CO_2_ at dark, the apoptotic cells were measured on a flow cytometer machine (BD Bioscience).

### 2.11 Western blot assay

All proteins were extracted from cells using RIPA lysis buffer (Beyotime Institute of Biotechnology). Then, the concentrations of proteins were measured by Bicinchoninic Acid Protein Assay Kit (Pierce Biotechnology, Inc.) following the protocol. Afterward, 20 μg proteins were separated by 10% SDS‐PAGE gel and transferred onto PVDF membranes. Membranes were blocked with 5% skimmed milk at room temperature for 50 min and incubated with primary antibodies at 4°C overnight. The primary antibodies were as follows: rabbit anti‐β‐catenin (ab32572; 1:5000; Abcam), rabbit anti‐TCF4 (ab217668; 1:10,000; Abcam), rabbit anti‐Cyclin D1 (ab16663; 1:100; Abcam), rabbit anti c‐Myc (ab32072; 1:1000; Abcam), and rabbit anti GAPDH (ab9485; 1:2500; Abcam). After that, membranes were washed with Tris‐buffered saline solution (containing 0.1% Tween‐20) and then incubated with goat anti‐rabbit IgG H&L secondary antibody (ab7090; 1:1000; Abcam) at room temperature for 50 min at dark. Next, proteins were detected with enhanced chemiluminescence (Pierce Biotechnology, Inc.) and analyzed using ImageJ 1.48 software (National Institutes of Health) while normalized to GAPDH.

### 2.12 Statistical analysis

The experiment data were expressed as mean ± standard deviation, and one‐way analysis of variance (ANOVA) was performed using the statistical software SPSS 16.0 software (IBM). ROC curves and Spearman's correlation test were used to analyze specific biomarkers’ diagnostic value and the correlation between two factors. Significant differences existed if *p* < 0.05 in statistical difference analysis.

## 3 RESULTS

### 3.1 LUCAT1 was upregulated in COPD

**FIGURE 1:**
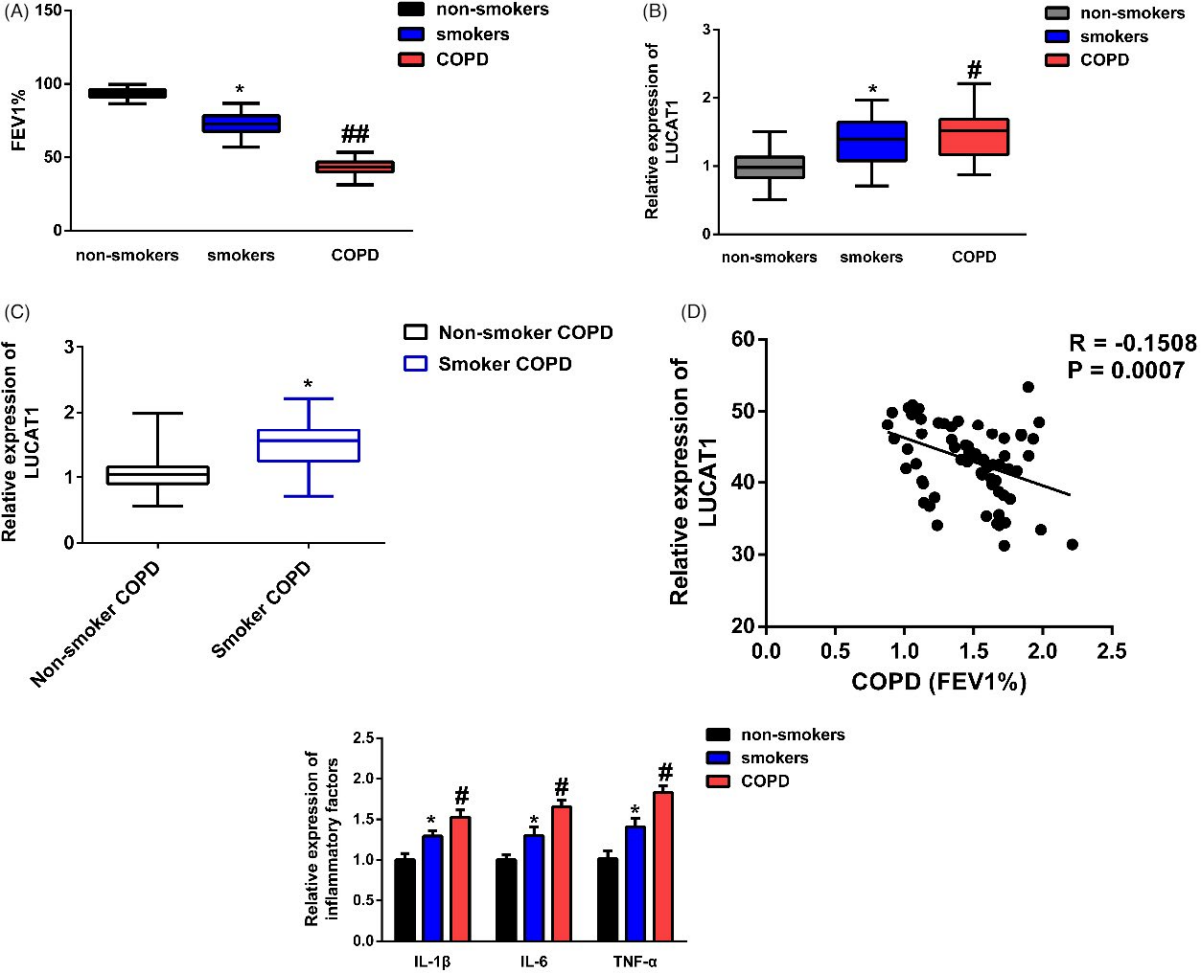
LUCAT1 expression was significantly increased in COPD patients. (A) FEV1% was decreased in smokers and COPD patients compared with non‐smokers. ^*^ *p* < 0.05, smokers vs. non‐smokers; ^##^ *p* < 0.01, COPD vs. smokers. (B) LUCAT1 was upregulated in smokers and COPD patients. ^*^ *p* < 0.05, smokers vs. non‐smokers; ^##^ *p* < 0.01, COPD vs. smokers. (C) Expression level of LUCAT1 in COPD with or without smoking. ^*^ *p* < 0.05, smoker COPD vs. non‐smoker COPD. (D) LUCAT1 expression was negatively correlated with FEV1%. (E) IL‐1β, IL‐6, and TNF‐α were increased in smokers and COPD patients. ^*^ *p* < 0.05, smokers vs. non‐smokers; ^#^ *p* < 0.05, COPD vs. smokers

The qRT‐PCR analysis was conducted to evaluate the expression of LUCAT1 expression in all subjects. From Figure 1A, through the pulmonary function test, we found that FEV1% was significantly downregulated in smokers and COPD patients than non‐smokers; meanwhile, the downregulation was more evident in the COPD group. Furthermore, Figure 1B revealed a notable increase in LUCAT1 expression in serum samples from smokers and COPD patients. Further, we divided COPD cases into non‐smoker COPD and smoker COPD. In Figure 1C, we found that LUCAT1 expression was significantly increased in smoker COPD group compared with non‐smoker COPD. Also, LUCAT1 expression was verified to be negatively correlated with FEV1% in COPD patients (*r* = −0.1508, Figure 1C). To determine the association between LUCAT1 expression and inflammation in COPD, an ELISA assay was conducted accordingly. As shown in Figure 1D and Table 1, IL‐1β, IL‐6, and TNF‐α were remarkably increased in smokers and COPD patients in relation to non‐smokers. LUCAT1 level was positively correlated with IL‐1β, IL‐6, and TNF‐α expression in COPD.

### 3.2 LUCAT1 functioned as a diagnostic marker in patients with COPD

**FIGURE 2:**
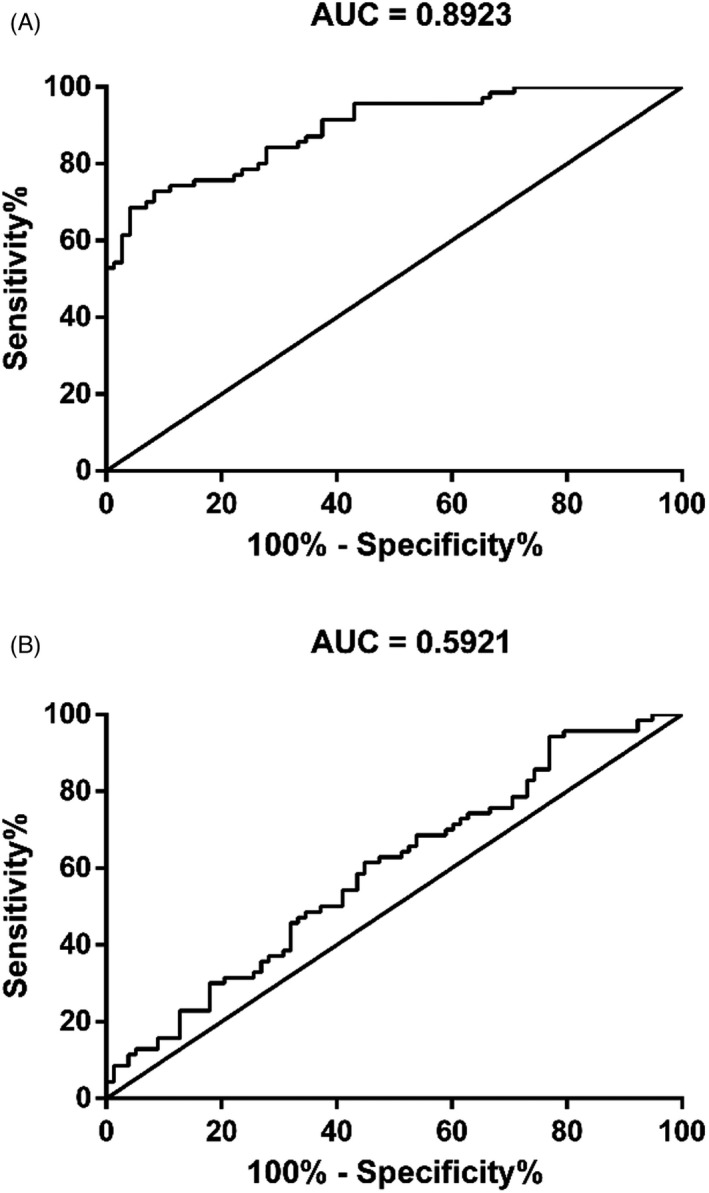
LUCAT1 was a potential diagnostic biomarker for COPD. (A) ROC analysis of LUCAT1 in differentiating COPD patients from non‐smokers. (B) ROC analysis of LUCAT1 in differentiating COPD patients from smokers

The potential of LUCAT1 in COPD clinical diagnosis was illustrated by ROC analysis. As demonstrated in Figure 2A, LUCAT1 could differentiate COPD from smokers with the area under the curve (AUC) of 0.8923. Moreover, LUCAT1 may also discriminating COPD from non‐smokers with high sensitivity, specificity, and accuracy as well (AUC = 0.5921).

### 3.3 LUCAT1 silencing alleviated CSE‐induced apoptosis and promoted proliferation in HBE cells

**FIGURE 3:**
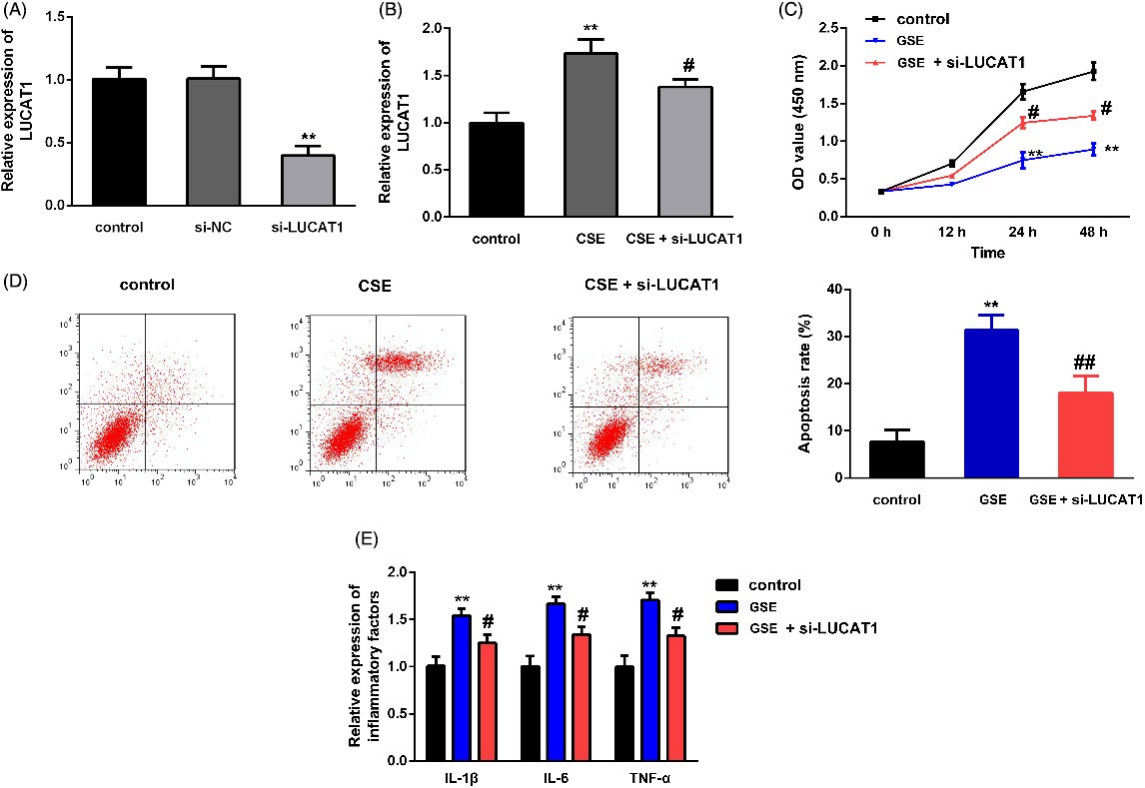
LUCAT1 silencing reversed the effect of CSE on 16HBE cell progression. (A) LUCAT1 was significantly downregulated in si‐LUCssAT1 group after transfection. ^**^ *p* < 0.05, si‐LUCAT1 vs. si‐NC. (B) CSE treatment increased LUCAT1 expression in 16HBE cells, and the variation could be partially counteracted by co‐transfection with si‐LUCAT1. ^**^ *p* < 0.01, CSE vs. control; ^#^ *p* < 0.05, CSE + si‐LUCAT1 vs. CSE. (C) LUCAT1 knockdown reversed the inhibition of CSE on 16HBE cell proliferation. ^**^ *p* < 0.01, CSE vs. control; ^#^ *p* < 0.05, CSE + si‐LUCAT1 vs. CSE. (D) LUCAT1 downregulation abolished the promotion of CSE on 16HBE cell apoptosis. ^**^ *p* < 0.01, CSE vs. control; ^##^ *p* < 0.01, CSE + si‐LUCAT1 vs. CSE. (E) LUCAT1 silencing counteracted the effects of CSE on inflammatory cytokines in 16HBE cells. ^**^ *p* < 0.01, CSE vs. control; ^#^ *p* < 0.05, CSE + si‐LUCAT1 vs. CSE

After constructing the LUCAT1 silencing plasmid, the transfection efficiency of LUCAT1 was detected. As shown in Figure 3A, the expression of LUCAT1 was markedly reduced in the si‐LUCAT1 group compared with the si‐NC group. Furthermore, after CSE treatment, we found that LUCAT1 level was increased; however, the increase could be partially inhibited by transfection with si‐LUCAT1 (Figure 3B). Besides, Figure 3C,D illustrated that 16HBE cell proliferation was inhibited and apoptosis was promoted after CSE treatment, whereas the inhibition of proliferation and promotion in apoptosis could be partially counteracted by transfection with si‐LUCAT1. It is more important that the inflammation in CSE‐treated 16HBE cells could be ameliorated by knocking down LUCAT1 expression as well (Figure 3E).

### 3.4 miR‐181a‐5p downregulation attenuated the effects of LUCAT1 knockdown on COPD progression

**FIGURE 4:**
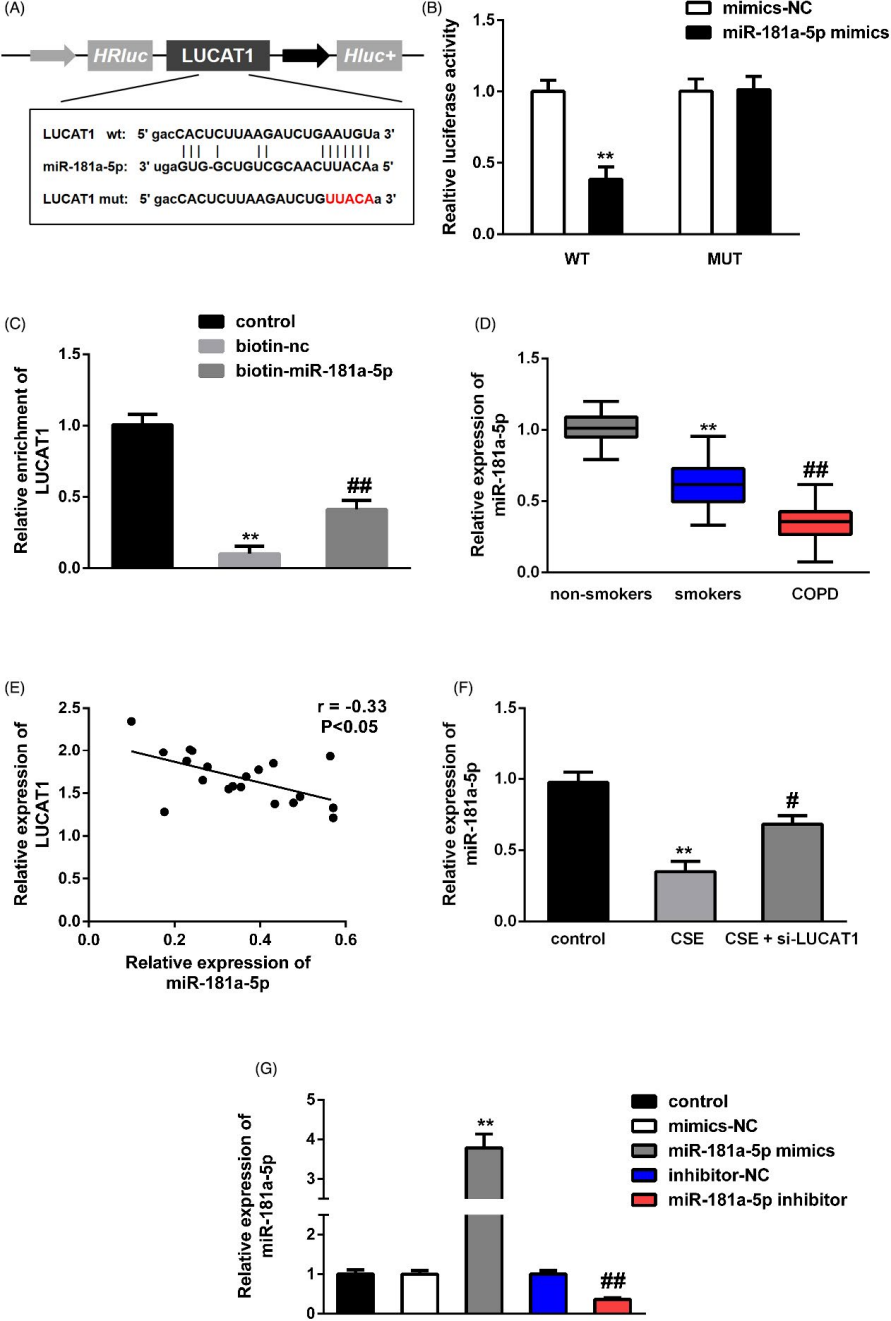
miR‐181a‐5p targeted LUCAT1. (A) The potential miR‐181a‐5p binding site in LUCAT1. (B, C) Dual‐luciferase reporter and RNA pull‐down assays confirmed the interaction between miR‐181a‐5p and LUCAT1. ^**^ *p* < 0.01, miR‐181a‐5p mimics vs. mimics‐NC; ^**^ *p* < 0.01, biotin‐nc vs. control; ^##^ *p* < 0.01, biotin‐miR‐181a‐5p vs. biotin‐nc. (D) miR‐181a‐5p expression in serum samples from COPD, non‐smokers and smokers. ^**^ *p* < 0.01, smokers vs. non‐smokers; ^##^ *p* < 0.01, COPD vs. smokers. (E) Correlation between miR‐181a‐5p and LUCAT1 in COPD patients. (F) LUCAT1 silencing increased miR‐181a‐5p expression after CSE treatment. ^**^ *p* < 0.01, CSE vs. control; ^#^ *p* < 0.05, CSE + si‐LUCAT1 vs. CSE. (G) miR‐181a‐5p expression was significantly increased in miR‐181a‐5p mimics group but decreased in miR‐181a‐5p inhibitor group after transfection. ^**^ *p* < 0.01, miR‐181a‐5p mimics vs. mimics‐NC; ^##^ *p* < 0.01, miR‐181a‐5p inhibitor vs. inhibitor‐NC

As to verify the correlation between miR‐181a‐5p and LUCAT1, dual‐luciferase reporter and RNA pull‐down assays were conducted subsequently. As illustrated in Figure 4A–C, miR‐181a‐5p was a potential target of LUCAT1. Furthermore, miR‐181a‐5p expression was prominently decreased in smokers and COPD patients; the variation was higher in the COPD group than in smokers (Figure 4D). Results in Figure 4E displayed a negative correlation between miR‐181a‐5p and LUCAT1 in COPD patients. Furthermore, miR‐181a‐5p expression was decreased after exposure with CSE; however, after co‐transfection with si‐LUCAT1, the miR‐181‐5p level was increased (Figure 4F,G).

### 3.5 The LUCAT1/miR‐181a‐5p/Wnt/β‐catenin axis in COPD

**FIGURE 5:**
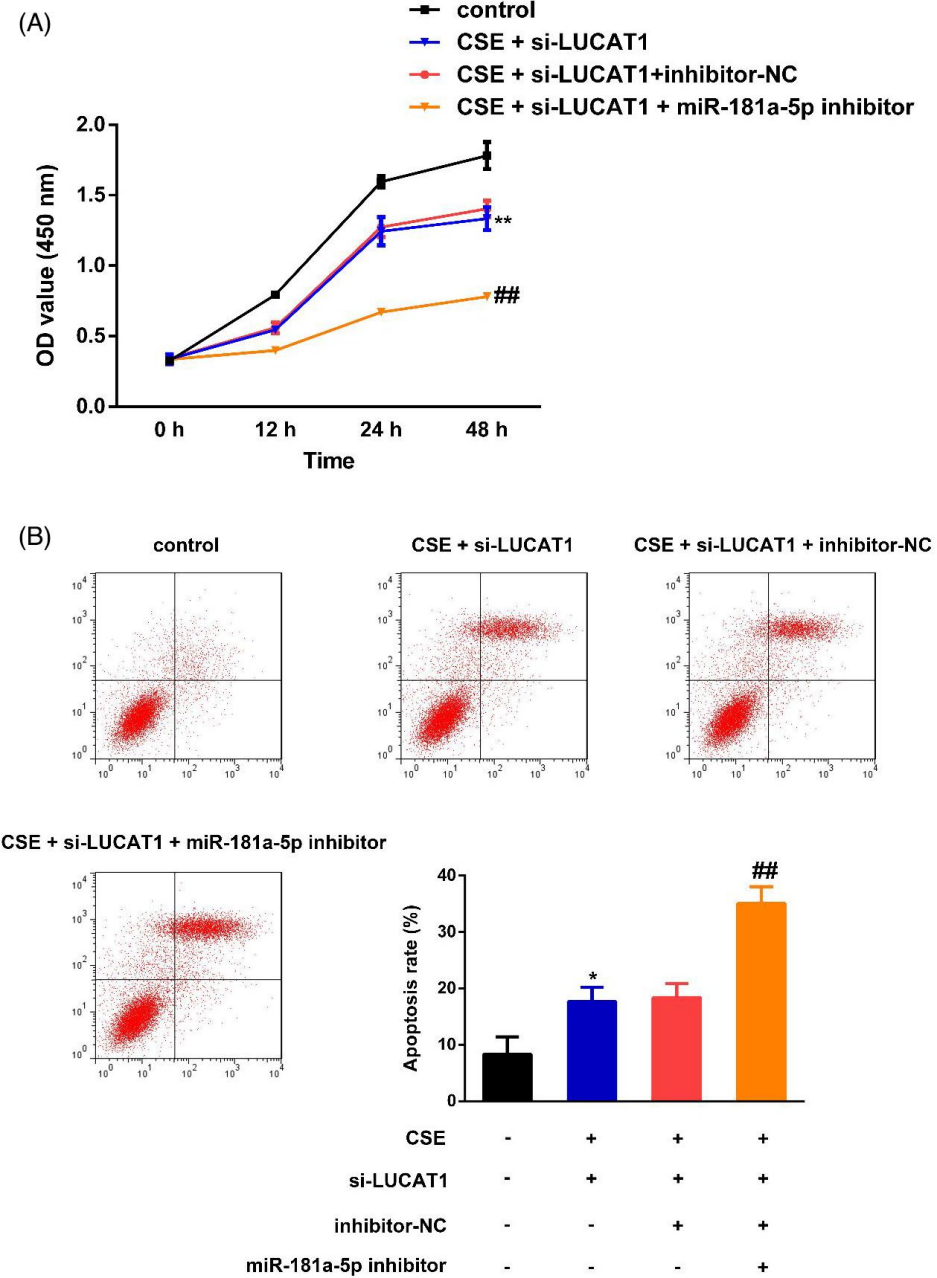
miR‐181a‐5p downregulation abolished the effects of LUCAT1 silencing on COPD progression. (A) miR‐181a‐5p inhibitor reversed the promotion of LUCAT1 silencing on CSE‐induced 16HBE cell proliferation. ^**^ *p* < 0.01, CSE + si‐LUCAT1 vs. control; ^##^ *p* < 0.01, CSE + si‐LUCAT1 + miR‐181a‐5p inhibitor vs. CSE + si‐LUCAT1. (B) miR‐181a‐5p inhibitor counteracted the inhibition of LUCAT1 knockdown on CSE‐induced 16HBE cell apoptosis. ^*^ *p* < 0.05, CSE + si‐LUCAT1 vs. control; ^##^ *p* < 0.01, CSE + si‐LUCAT1 + miR‐181a‐5p inhibitor vs. CSE + si‐LUCAT1

**FIGURE 6:**
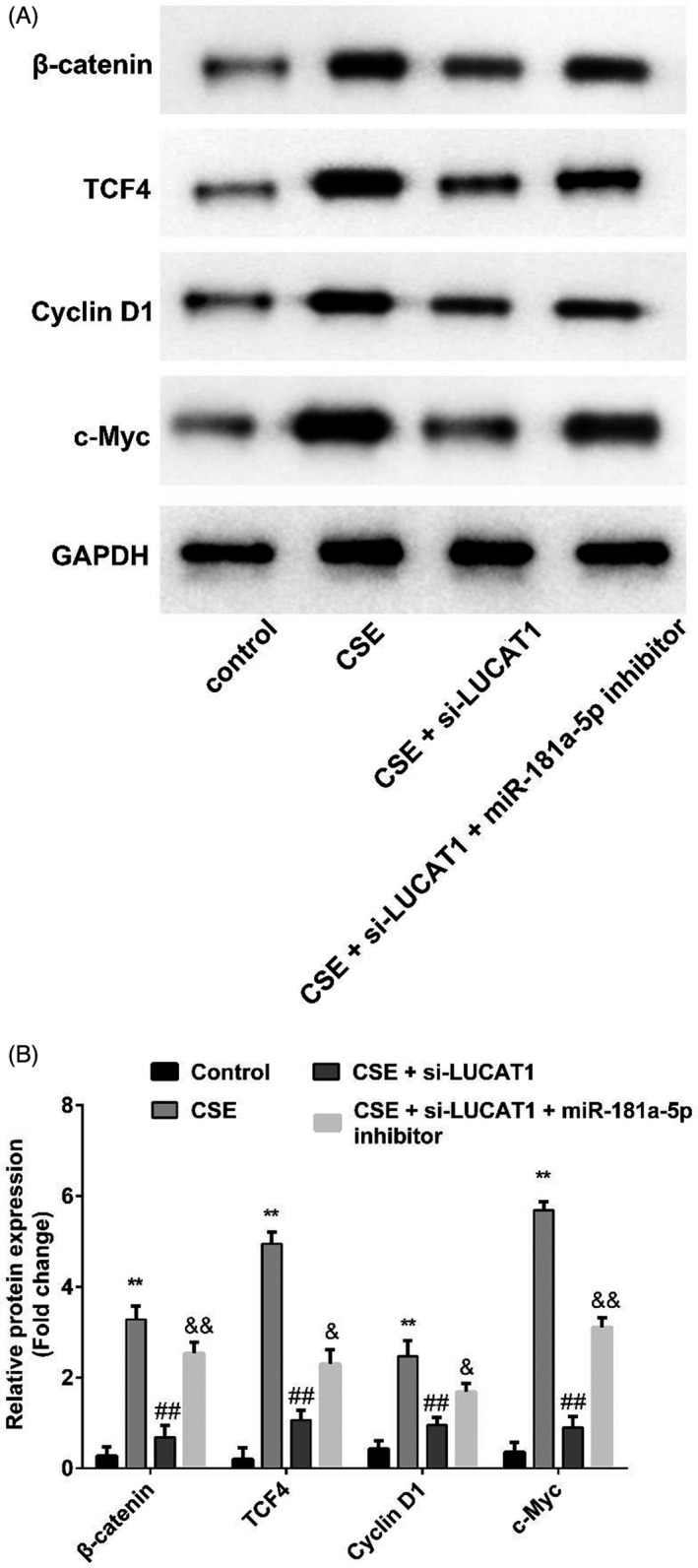
LUCAT1/miR‐181a‐5p/Wnt/β‐catenin signaling pathway in COPD. (A) Western blot assay revealed the expressions of β‐catenin, TCF4, Cyclin D1, and c‐Myc in CSE‐treated 16HBE cells. (B) Quantified results of A were presented. ^**^ *p* < 0.01, CSE vs. control; ^##^ *p* < 0.01, CSE + si‐LUCAT1 vs. CSE; ^&^ *p* < 0.05, ^&&^ *p* < 0.01, CSE + si‐LUCAT1 + miR‐181a‐5p inhibitor vs. CSE + si‐LUCAT1

As demonstrated in Figure 5A,B, we found that co‐transfection with miR‐181a‐5p inhibitor could abolish LUCAT1 knockdown effects on CSE‐induced 16HBE cell proliferation and apoptosis. Furthermore, Western blot in Figure 6A and B revealed that β‐catenin, TCF4, Cyclin D1, and c‐Myc levels were increased after CSE treatment. The increase could be prominently hindered by knocking down LUCAT1 expression in 16HBE cells. However, the miR‐181a‐5p inhibitor could also reverse the effects of LUCAT1 silencing on β‐catenin, TCF4, Cyclin D1, and c‐Myc levels.

## 4 DISCUSSION

With the continuous development of circulating lncRNA detection technologies, serum lncRNAs have received much attention due to their easy availability, long‐term stability, and non‐invasive advantages. For example, it has been reported that the levels of lncRNA PFRL^16^ and lncRNA MEG3^17^ were significantly elevated in the patients with idiopathic pulmonary fibrosis (IPF) and can be used as potential prognostic indicators. Moreover, LncRNA LUCAT1 expression was significantly increased in various cancers, such as liver cancer,^18^ triple‐negative breast cancer,^19^ gastric cancer,^20^ colorectal cancer,^21^ cervical cancer,^22^ hepatocellular carcinoma,^23^ and glioma,^24^ serving as a valuable biomarker in clinical treatment. In this study, we conducted an epidemiological investigation of 245 subjects. Serum LUCAT1 levels were measured in three cohorts of non‐smokers, non‐COPD smokers, and COPD, and the correlation between changes in serum LUCAT1 levels and inflammation was analyzed. Recently, Zhou et al. illustrated that lncRNA LUCAT1 was significantly upregulated in tissues from COPD patients using microarray analysis. However, Zhou et al.^25^ did not study the underlying mechanisms of LUCAT1 in COPD development. Based on previous study, we conducted this study. Consistent with this study,^25^ the results in our study showed that serum LUCAT1 levels were increased in smokers without COPD and in patients with COPD compared with non‐smokers; simultaneously, the difference in LUCAT1 level was more significant in patients with COPD and smoker COPD group, suggesting that COPD disease status and smoking may cause elevated serum LUCAT1 levels. It is more important that the ROC analysis confirmed the potential of LUCAT1 in discriminating COPD from smokers or non‐smokers, with the AUC results of 0.5921 and 0.8923. Moreover, our study's pulmonary function tests showed that the FEV1% in COPD and smokers without COPD was significantly reduced after inhaling salbutamol aerosol compared with non‐smokers, suggesting that long‐term smoking may cause a decline in lung function.

Smoking induced significant inflammation in the lungs.^26^ Smokers were found to have dose‐dependently elevated levels of inflammatory cells and inflammatory factors in BALF compared with non‐smokers, suggesting that the risk of smoking‐related inflammation increased with smoking intensity.^27^ It was found that smokers without COPD had an increased number of inflammatory cells in the bronchial mucosa, and decreased airway epithelial integrity.^28^, ^29^ Thus, CSE exposure might cause respiratory symptoms and lung injury, leading to low health quality. Previous studies elucidated that smoking induced the release of inflammatory factors such as IL‐1β, IL‐6, and TNF‐α, suggesting that inflammatory cytokines could play critical roles in mediating COPD’s acute onset and progression.^30^, ^31^, ^32^ In the present study, we established an in vitro model of COPD by exposing 16HBE cells to CSE. As pro‐inflammatory factors, IL‐1β, IL‐6, and TNF‐α were found to be elevated in smokers, COPD patients, and 16HBE cells after CSE treatment. LUCAT1 expression was strongly correlated with inflammatory cytokines expressions; meanwhile, LUCAT1 silencing could also lead to decreased IL‐1β, IL‐6, and TNF‐α as well. Moreover, LUCAT1 knockdown could promote CSE‐induced 16HBE cell proliferation but inhibit apoptosis as well. Mechanically, miR‐181a‐5p was proved to be a target of LUCAT1. The negative relationship between miR‐181a‐5p and LUCAT1 in COPD was confirmed by qRT‐PCR and Spearman's correlation analysis. Collectively, LUCAT1 expression was related to inflammation in COPD, exerting its function in COPD diagnosis.

Wnt/β‐catenin was a widespread transcription factor regulating a number of genes critical for immunity, cell growth, inflammation, and tumor development.^33^, ^34^, ^35^, ^36^ Activation of Wnt/β‐catenin has been detected in COPD patients as well.^37^, ^38^, ^39^ It was accepted that COPD was associated with the production of several inflammatory factors and chemokines, including IL‐1β, IL‐6, and TNF‐α.^30^, ^31^, ^32^ Other studies have found that both TNF‐α, IL‐1β, and IL‐6 could induce proliferation and apoptosis of airway epithelial cells through activation of Wnt/β‐catenin.^40^, ^41^, ^42^ These pieces of evidence suggested that the Wnt/β‐catenin pathway played a crucial role in chronic lung disease. Western blot results in our study suggested that LUCAT1 silencing promoted TCF4, Cyclin D1, c‐Myc, and β‐catenin expressions in CSE‐induced 16HBE cells; however, co‐transfection with miR‐181a‐5p inhibitor could partially reverse the variation in TCF4, Cyclin D1, c‐Myc, and β‐catenin induced by LUCAT1 silencing. Collectively, our data presented that LUCAT1 may regulate CSE‐induced 16HBE cell proliferation and apoptosis by targeting miR‐181a‐5p through the Wnt/β‐catenin pathway miR‐181a‐5p had a feedback effect on LUCAT1 as well.

Taken together, our results elucidated the potential value of the LUCAT1‐miR‐181a‐5p‐ Wnt/β‐catenin axis on the progression of COPD, providing new sights for treatment.

## CONFLICT OF INTEREST

None.

## Data Availability

The datasets used and/or analyzed during the current study are available from the corresponding author on reasonable request.
